# Development and Validation of an UPLC–MS/MS Method for Remodelin Quantification in Mouse Plasma and Tissues: Application to Biodistribution and Ultrastructural Assessment

**DOI:** 10.1155/jamc/9654340

**Published:** 2026-08-24

**Authors:** Litao Wang, Quanyu Liu, Cheng Yu, Yu Zhong, Minxia Wu, Xi Lin, Yan Hu

**Affiliations:** ^1^ The Graduate School of Fujian Medical University, Fuzhou 350004, China, fjmu.edu.cn; ^2^ Department of Pharmacy, Fujian Health College, Fuzhou 350101, China, fjwx.com.cn; ^3^ Department of Cardiology, Fujian Medical Center for Cardiovascular Diseases, Fujian Institute of Coronary Heart Disease, Fujian Medical University Union Hospital, Fuzhou 350000, China, fjmu.edu.cn; ^4^ The School of Pharmacy, Fujian Medical University, Fuzhou 350004, China, fjmu.edu.cn; ^5^ Public Technology Service Center, Fujian Medical University, Fuzhou 350004, China, fjmu.edu.cn

**Keywords:** bioanalytical validation, biodistribution, cell ultrastructure, remodelin, UPLC–MS/MS

## Abstract

Remodelin, a selective N‐acetyltransferase 10 (NAT10) inhibitor, has emerged as a promising therapeutic candidate for degenerative diseases with nuclear abnormalities and acetylation dysregulation. However, its pharmacological development has been hindered by the lack of a sufficiently sensitive bioanalytical method. To address this limitation, a simple, sensitive, and reliable ultra‐performance liquid chromatography coupled to tandem mass spectrometry (UPLC–MS/MS) method was established and fully validated. This method achieved accurate quantification of remodelin at low pg/mL levels, representing a significant improvement in sensitivity and excellent linearity (*R* ^2^ > 0.99). All validation parameters met the predefined bioanalytical acceptance criteria. This method was applied to quantify remodelin in C57BL/6J mouse plasma and various tissues (carotid artery, heart, liver, and kidney), with higher concentrations observed in the liver and kidney. Given the proposed role of remodelin in maintaining nuclear integrity, its protective effect was further investigated by transmission electron microscopy (TEM). The analysis provided direct evidence of a protective effect on nuclear morphology, with treated tissues maintaining intact nuclear membranes and clear cellular architecture, whereas untreated tissues exhibited varying degrees of structural damage. These findings established a robust analytical method for remodelin quantification and provided preliminary ultrastructural evidence of its protective effects, supporting further pharmacodynamic investigation.

## 1. Introduction

N‐Acetyltransferase 10 (NAT10) is a nucleolar protein composed of 1025 amino acids with a molecular weight of approximately 116 kDa. Its structure consists of a tRNA‐binding domain and an RNA de‐conjugase domain [1]. As the sole discovered modifying enzyme (writer) connected to N4‐acetylcytidine (ac4C) modification [2], NAT10 catalyzes this post‐transcriptional modification to enhance mRNA stability and translational efficiency [3]. The acetyltransferase modifies various substrates, such as histones, microtubules, tRNA, or mRNA. Beyond its enzymatic functions, it is involved in critical cellular processes, like mitotic cytokinesis and stress response pathways. NAT10 is overexpressed in numerous human malignancies, where its elevated levels promote cancer cell proliferation, migration, and invasion [4–9]. In addition to its role in cancer, NAT10 is also involved in the occurrence of non‐neoplastic diseases, most notably Hutchinson–Gilford progeria syndrome (HGPS) [10] and vascular remodeling [11], which are of particular relevance to the present study. Beyond these, NAT10 has also been implicated in metabolic dysregulation and aberrant bone homeostasis [12, 13].

Remodelin, a small molecule inhibitor, has attracted great attention in biomedical research due to its specific inhibition of NAT10 activity [14, 15]. By targeting NAT10, remodelin exerts its effects by modulating the acetylation of histones and microtubules. This modulation helps in reshaping the nucleus and reducing DNA damage signals, thereby enhancing overall nuclear health and function [16]. Additionally, by hindering cell proliferation and motility, remodelin restrains the migration and invasion of cancer cells, ultimately resulting in cell cycle arrest or apoptosis [17, 18]. Owing to its effective inhibition of NAT10 activity, remodelin demonstrates multifaceted therapeutic potential, including antitumor effects [19], healthspan enhancement in a mouse model of HGPS [20], and amelioration of non‐neoplastic disorders [21]. Given these broad pharmacological properties, remodelin is emerging as a promising therapeutic candidate [22].

Despite this progress, the pharmacological development has been constrained by two major gaps: one is the lack of a sufficiently sensitive bioanalytical method to study its biodistribution in plasma and various tissues, and the other is the limited understanding of its impact on cellular ultrastructure, particularly nuclear morphology in tissues following administration. Precise quantification of remodelin in biological samples is essential for advancing its clinical translation. Moreover, detailed ultrastructural analyses of nuclear remodeling could yield fundamental insights into remodelin’s impact on nuclear architecture and its consequences on cellular organization. Therefore, accurately determining its concentration in biological samples and observing its influence on cell ultrastructure are of great significance for understanding its pharmacological effects and advancing clinical translation.

Ultra‐high performance liquid chromatography–tandem mass spectrometry (UPLC–MS/MS) has become a preferred technique for quantifying analytes due to its high speed, superior sensitivity, outstanding specificity, and capability to analyze complex biological samples [23]. Compared with traditional methods, UPLC–MS/MS gives better separation and higher sensitivity. The short run time allows fast analysis of many samples, which is important for tissue distribution studies [24–26]. These features make UPLC–MS/MS well suited for measuring small‐molecule drugs like remodelin in biological samples. The purpose of this study was to establish and fully validate a simple, sensitive, and reliable UPLC–MS/MS method for quantifying the concentration of remodelin in biological samples. The method was capable of accurately quantifying remodelin at low pg/mL levels, with all validation parameters meeting predefined bioanalytical acceptance criteria. Furthermore, the method was successfully applied to analyze the biodistribution of remodelin at 1 h following intraperitoneal administration. To verify the protective effect of remodelin, transmission electron microscopy (TEM) was utilized to examine ultrastructural changes in cellular architecture in different tissues pre‐ and post‐treatment with remodelin. The integrated results from UPLC–MS/MS quantification and TEM ultrastructural analysis provide compelling preclinical evidence, supporting the further development of remodelin as a promising therapeutic candidate [32].

## 2. Experimental Section

### 2.1. Chemicals and Reagents

Remodelin hydrobromide and methotrexate (MTX; the internal standard, IS) (both with purity > 99.9%) were obtained from MedChemExpress Biotechnology Co., Ltd. (Chengdu, China). HPLC‐grade methanol was purchased from Supelco (Shanghai, China), formic acid and dimethyl sulfoxide (DMSO) were bought from Macklin (Shanghai, China). Osmium tetroxide (OsO_4_) was provided by Alfa Aesar (Shanghai, China), UranyLess TEM staining solution was supplied by Micro to Nano (Haarlem, Netherlands). Water was purified through a Labonova Smart Water Purification System (Think‐lab Corporation, Boston, MA, USA). All other chemicals and reagents were of analytical grade and purchased from certified vendors.

### 2.2. Instrumentation and LC–MS/MS Conditions

UPLC–MS/MS analysis was performed on a 1290 Infinity LC system coupled with a 6410 triple quadrupole mass spectrometer, with data acquisition controlled by Masshunter Workstation Software Version B.08.00 (Agilent Technologies, Santa Clara, CA, USA).

Chromatographic separation was carried out on an ACQUITY UPLC BEH C18 column (2.1 mm × 50 mm, 1.7 μm; Waters, Wexford, Ireland). Mobile phase was composed of 0.1% formic acid (A) and methanol (B) with gradient elution at a flow rate of 0.3 mL/min. The gradient elution conditions were presented in Table 1. The gradient was optimized to balance resolution and throughput (5.5 min), with the initial 30% organic phase enabling rapid elution of polar matrix components and the ramp to 95% methanol ensuring sufficient column washing. The sample injection volume was 2.0 μL. The column temperature was maintained at 35°C.

**TABLE 1:** Gradient elution for chromatographic conditions.

t/min	Flow rate (mL/min)	A (%, v/v)^a^	B (%, v/v)^b^
Initial	0.3	70	30
0.50	0.3	5	95
3.00	0.3	5	95
3.01	0.3	70	30
5.50	0.3	70	30

^a^Water containing 0.1% formic acid.

^b^Methanol.

The mass spectrometer was operated with an electrospray ionization source in positive ion mode for remodelin and IS. Nitrogen for MS was supplied by the SF4FF nitrogen generator (Atlas Copco Corporation, Palmer, PA, USA). The main MS parameters for remodelin and IS were displayed in Table 2. Other optimized MS parameters were as follows: ion source temperature was set to 325°C, nebulizer gas to 40 psi, and ionization voltage to 3.5 kV. High‐purity nitrogen was used as a collision gas.

**TABLE 2:** Optimized mass parameters for remodelin and IS.

Analyte	Rt (min)	Precursor ion (*m*/*z*)	Product ion (*m*/*z*)	Fragmentor/V	Collision energy/eV
Remodelin	2.77	283.1	84.1^a^	105	15
		283.1	199.9^b^	105	20

Methotrexate	0.71	455.2	308.1^a^	95	22
		455.2	175.2^b^	95	35

^a^Ion for quantification.

^b^Ion for qualification.

### 2.3. Animals

Approximately 8‐ to 10‐week‐old male C57BL/6J mice, with an average body weight of 20 g, were obtained from the Animal Experimental Center of Fujian Medical University. All animal care and experimental procedures were conducted in accordance with protocols approved by the Animal Care Committee of Fujian Medical University, with the approval number: IACUC FJMU 2022‐0704. All animals were kept in a temperature‐controlled environment (21°C–22°C) with standardized lighting conditions (12 h light/12 h dark).

### 2.4. Preparation of Stock Solutions, Calibration Standards, and Quality Control (QC) Samples

Stock powder of remodelin was ultrasonically dissolved in methanol to obtain a final concentration of 1.0 mg/mL. IS stock solution was similarly prepared by dissolving the powder in a methanol solution containing 3.3% DMSO, achieving a final concentration of 1.0 mg/mL. Aliquots of stock solution were stored at −40°C.

The stock solution for plasma, carotid artery, and heart was progressively diluted using 50% methanol to prepare working solutions (0.61, 4.88, 19.53, 39.06, 312.5, 625, 2500, and 10,000 ng/mL), as well as QC working solutions (1.8, 5000, and 7500 ng/mL). All working solutions were stored at −40°C. Standard curve and QC samples were prepared by spiking 10 μL of the respective working solutions into 90 μL of blank plasma, carotid artery, or heart homogenate. The final concentrations of the standard curve samples were 0.06, 0.49, 1.95, 3.91, 31.25, 62.5, 250, and 1000 ng/mL, while the IS concentration in all samples was 100 ng/mL (10 μL added). The QC samples were prepared at final concentrations of 0.18 ng/mL (LQC), 500 ng/mL (MQC), and 750 ng/mL (HQC).

The stock solution of remodelin was sequentially diluted with 50% methanol to prepare working solutions for liver (2.44, 9.76, 19.53, 78.12, 312.5, 1250, 5000, and 20,000 ng/mL) and kidney (3.05, 12.21, 24.41, 97.66, 781.25, 3125.0, 12,500, and 50,000 ng/mL). QC working solutions for liver were prepared at concentrations of 7.2 ng/mL (LQC), 10,000 ng/mL (MQC), and 15,000 ng/mL (HQC), while those for kidney were prepared at 10 ng/mL (LQC), 25,000 ng/mL (MQC), and 37,500 ng/mL (HQC). The standard curve samples and QC samples of liver and kidney were processed the same as those of plasma, carotid artery, and heart. These matrix‐specific calibration ranges and QC levels were established based on expected concentration differences from preliminary data, ensuring accurate quantification within the validated linear dynamic range for each matrix.

Of note, the above QC concentrations were used for the assessment of intra‐day and inter‐day accuracy and precision. For recovery, matrix effect, and stability evaluations, a separate set of QC samples prepared at concentrations of 0.24 ng/mL (LQC), 15.62 ng/mL (MQC), and 500 ng/mL (HQC) for plasma, carotid artery, and heart; 0.488 ng/mL (LQC), 15.625 ng/mL (MQC), and 1000 ng/mL (HQC) for liver; and 0.61 ng/mL (LQC), 39.062 ng/mL (MQC), and 2500 ng/mL (HQC) for kidney were used. Recovery, matrix effect, and stability are primarily governed by the extraction procedure, matrix composition, and storage conditions and are independent of the specific concentration levels within the validated linear range.

### 2.5. Sample Preparation

Approximately 100 mg of frozen tissue samples were left to thaw at room temperature. Subsequently, samples were homogenized in 300 μL saline via bead beating, followed by centrifugation at 12,000 rpm for 15 min to collect the supernatant. In a 1.5 mL centrifuge tube, 10 μL of a 100 ng/mL IS solution was added to 100 μL of plasma or tissue samples. Protein precipitation was then performed by adding 400 μL of methanol and thoroughly mixing on a vortex mixer for 3 min, after which the mixture was centrifuged at 12,000 g at 4°C for an additional 10 min. The resulting supernatant was transferred to a new tube and subjected to another round of centrifugation under the same condition (12,000 g at 4°C for 10 min). Following this step, the supernatant was carefully transferred into the inner tube of a sample vial. The plasma was processed by a simple protein precipitation method using methanol, and the volume ratio of plasma to final volume was 1:5. Finally, both treated plasma and tissue samples were chromatographically separated and detected via UPLC–MS/MS.

### 2.6. Method Validation

The analytical method was validated in accordance with the U.S. Food and Drug Administration (FDA, 2018) bioanalytical guidelines [27]. The validation parameters included selectivity, linearity, LLOQ, accuracy, precision, matrix effect, recovery, and stability [28–30].

#### 2.6.1. Selectivity

Six different sources of blank mouse plasma or tissues, blank mouse plasma or tissues spiked with remodelin at LLOQ and IS, and the plasma or tissue samples after intraperitoneal administration of remodelin were analyzed by UPLC–MS/MS to determine if endogenous matrix components in blank plasma or tissue homogenates affected the analytical specificity of remodelin quantification.

#### 2.6.2. Linearity and LLOQ

Calibration curves were constructed by plotting peak area ratios of remodelin to IS versus the concentrations of the standard curve samples. An 8‐point calibration curves were constructed using matrix‐matched standards (plasma and tissue homogenates), with each concentration level analyzed in triplicate (*n* = 3). Linear regression of each calibration curve was performed using a standard 1/x (x = nominal concentration) weighting option to aid in covering a wide dynamic range.

The LLOQ was defined as the lowest concentration that could be quantified with a signal‐to‐noise ratio (S/N) exceeding 10 and with both accuracy and precision no more than 20%.

#### 2.6.3. Carryover

Carryover was evaluated by injecting a blank matrix sample immediately after the highest quantification standard in three independent analytical runs. The peak area of remodelin in the blank injections was compared to that of the LLOQ sample to determine the potential carryover percentage.

#### 2.6.4. Accuracy and Precision

Accuracy and precision, both intra‐ and inter‐day, were evaluated through three independent analytical runs of QC samples at three concentration levels (LQC, MQC, HQC) with five replicates. Accuracy and precision were expressed as relative error (RE) and relative standard deviation (RSD), respectively. RE was calculated as [(mean back‐calculated concentration − nominal concentration)/nominal concentration] × 100%, while RSD was calculated as (standard deviation/mean measured concentration) × 100%. The acceptance criteria for RSD and RE were set at ±15%.

#### 2.6.5. Recovery and Matrix Effect

Recovery rates were determined by comparing the peak area ratios of the remodelin to the IS before and after sample processing at three concentration levels (LQC, MQC, HQC; *n* = 5 each). The matrix effect was investigated by calculating the ratios of the peak area in the presence of the matrix to the peak area in the absence of the matrix.

#### 2.6.6. Stability

To assess the stability of plasma and tissue samples in the short and long term, QC samples were examined under different storage conditions (*n* = 5 replicates/condition). Condition 1 was placed at room temperature for 4 h; condition 2 was stored at 4°C for 24 h; condition 3 underwent three complete freeze‐thaw cycles (from −40°C to room temperature). The long‐term stability of remodelin was evaluated by analyzing the QC samples stored at −40°C for 7 days, with acceptance criteria set at ±15% for both RSD and RE.

### 2.7. Quantitative Analysis of Remodelin in Biological Samples

Six male C57BL/6J mice were subjected to wire‐induced carotid artery injury, followed by intraperitoneal administration of remodelin at a dose of 5 mg/kg/day. After 28 days of treatment, the mice were euthanized at 1 h after the final dose, and then the plasma, carotid artery, heart, liver, and kidney were collected. The tissues were rinsed with ice‐cold 0.9% saline, excess fluid was blotted, and samples were accurately weighed before being stored at −40°C. The sample preparation was performed as described in Section Sample Preparation.

### 2.8. Ultrastructural Analysis of Cells

The ultrastructure characteristics of different tissues were analyzed using TEM. First, the carotid arteries, hearts, livers, and kidneys from different mice after one month of treatment with either remodelin or DMSO were rapidly dissected into approximately 1 cm^3^ sections, then washed 3 times in phosphate‐buffered saline (PBS). Second, the tissues were immediately fixed overnight at 4°C in a solution containing 3% glutaraldehyde–1.5% paraformaldehyde in 0.1 M phosphate buffer (pH 7.2), followed by thorough washing at least three times for 10 min each in PBS to remove excess fixative. Third, samples were post‐fixed with 1% OsO_4_–1.5% K_4_Fe(CN)_6_ for 2 h at 4°C, then washed thoroughly as above. Fourth, they were dehydrated sequentially in 50% ethanol and 70% ethanol for 10 min each, then transferred into 70% ethanol‐saturated uranyl acetate solution overnight at 4°C. Fifth, after removing the uranyl acetate solution, samples were dehydrated through an ascending ethanol series (90% and 100%, 10 min each), followed by three 10 min washes in anhydrous acetone. Sixth, samples were infiltrated with graded acetone and 618 epoxy resin mixtures (volume ratio of acetone vs. 618 = 3:1, 1:1, 1:3) for 1 h each. After infiltration, samples were embedded in 100% 618 epoxy resin with stepwise polymerization: 12 h at 35°C, 12 h at 45°C, and 72 h at 60°C. Seventh, ultrathin sections (approximately 90‐nm thick) were obtained using an EM UC7 ultramicrotome (Leica, Vienna, Austria), placed on 200‐mesh copper grids, followed by staining with uranyl acetate substitute (UranyLess) and lead citrate for 8 min each [31]. Finally, the specimens were examined under a Tecnai G2 TEM (FEI, Hillsboro, OR, USA) operated at 120 kV. All the samples of different tissues were collected from two independent experiments with at least three biological replications.

## 3. Results and Discussion

### 3.1. Analytical Method Optimization

Chromatographic and mass spectrometric conditions were optimized to enhance sensitivity and resolution. The optimization of chromatographic conditions included column type, flow rate, and mobile phase composition, aiming to achieve sufficient peak response within a shorter run time. The ACQUITY UPLC BEH C18 column (details provided in Section Instrumentation and LC–MS/MS conditions) showed faster separation speed and higher peak capacity than the other columns screened, namely, two ZORBAX RR Eclipse Plus C18 columns (2.1 × 50 mm and 2.1 × 100 mm, 3.5 μm), and one ZORBAX RRHT StableBond C18 column (2.1 × 50 mm, 1.8 μm). To minimize the interference from impurities in biological samples, a linear gradient elution program was employed. Using a mobile phase of methanol/water modified with formic acid, remodelin and IS were eluted at different flow rates (0.2–0.4 mL/min) and varying formic acid concentrations (0.05%, 0.1%, and 0.2%). We found that remodelin exhibited the maximum signal intensity at a flow rate of 0.3 mL/min. Considering the compound’s susceptibility to decomposition under acidic conditions, a formic acid concentration of 0.1% was ultimately selected, despite the peak area reaching its maximum at a formic acid concentration of 0.2%.

**FIGURE 1:**
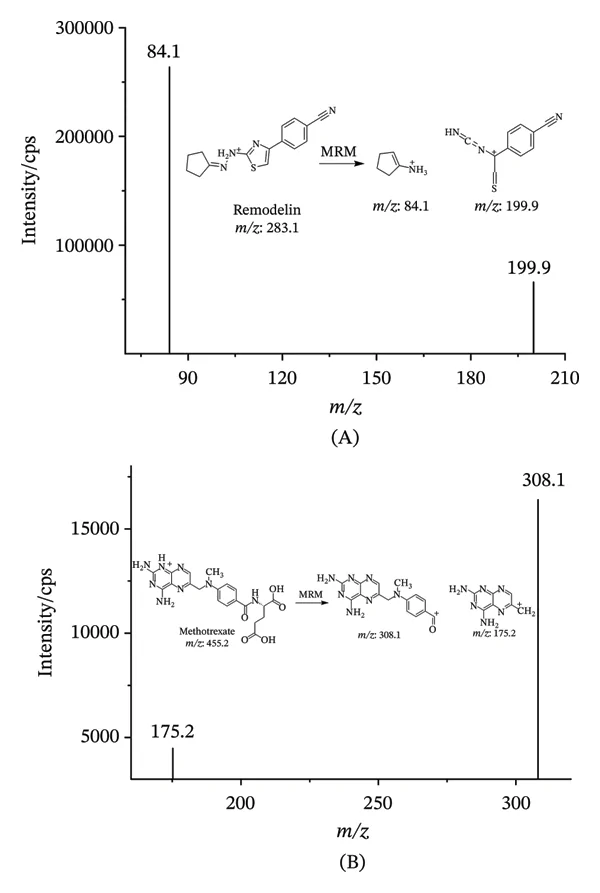
Mass spectra and proposed fragmentation of (A) remodelin and (B) IS.

The remodelin compound features a hydrazone group (*N–N=C*), whose nitrogen atom is strongly basic and readily accepts protons (H^+^), making it ideal for positive‐ion mode mass spectrometry analysis. The protonation of the hydrazone group was enhanced when formic acid was added to the mobile phase, thereby improving ionization efficiency and signal sensitivity. As for IS, the formic acid also promoted its protonation at the available basic nitrogen sites. MTX was selected as the IS because a stable isotope‐labeled remodelin analog was commercially unavailable. MTX shares a similar basic nitrogen‐containing heterocyclic structure with remodelin and exhibits stable ionization under the same positive‐ion mode conditions, making it a suitable structural alternative. During the characterization of remodelin’s fragment ions, the most abundant peak was observed at *m/z* 84.1. Therefore, the transition *m/z* 283.1 ⟶ *m/z* 84.1 was selected as the quantifier ion transition for MRM‐based quantification, while the transition *m/z* 283.1 ⟶ *m/z* 199.9 served as a qualifier ion transition. Although the fragment ion at *m/z* 199.9 was not of the highest abundance, its formation involved a multistep fragmentation pathway (including bond cleavage, ring‐opening, and rearrangement) rather than a simple direct bond cleavage. These fragmentation processes resulted in the formation of a conjugated system, which stabilized the product ion. For the IS, the transition *m/z* 455.2 ⟶ *m/z* 308.1 was monitored. The molecular structures and mass spectra of remodelin and IS, along with its characteristic product ions, were illustrated in Figure 1.

### 3.2. Method Validation

**FIGURE 2:**
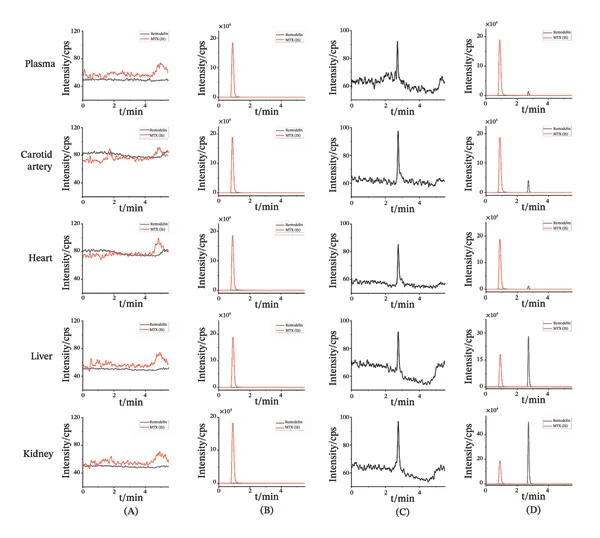
MRM chromatograms of remodelin and IS in biological samples, (A) blank; (B) blank spiked with remodelin at LLOQ and IS; (C) extract of the MRM chromatograms of remodelin at LLOQ from (B); and (D) biological samples after intraperitoneal administration of remodelin.

In the presence of target and nontarget components, an appropriate analytical method should be able to unequivocally measure the concentration of the analytes, which is termed the selectivity of a method. According to validation guidelines, the response in the blank should not exceed 20% of the LLOQ for the analytes. The typical MRM chromatograms of blank mouse plasma or tissues, blank mouse plasma or tissues spiked with remodelin at LLOQ and IS, and plasma samples or tissues after intraperitoneal administration of remodelin were shown in Figure 2. The chromatographic analysis showed that remodelin eluted at 2.77 min, while the IS was at 0.71 min (*n* = 5), with no co‐eluting interference peaks. The difference in retention time (Rt) between MTX and remodelin is primarily attributable to their distinct polarities: MTX, with multiple carboxyl and amino groups, is highly hydrophilic and thus elutes much earlier under the starting mobile phase conditions (30% organic phase, 70% aqueous). In contrast, remodelin, being less polar, is more strongly retained on the C18 column, resulting in a later Rt. Although MTX elutes earlier than remodelin, this Rt gap was considered acceptable because matrix‐effect compensation was thoroughly evaluated during validation using QC samples across all matrices. The selectivity test indicated that the UPLC–MS/MS assay had high specificity for detecting remodelin in mouse biological samples.

Table 3 summarized the regression equations, correlation of coefficients (*R* ^2^) for the quantification of remodelin in plasma and different tissues. All the calibration curves demonstrated excellent linearity, with *R* ^2^ greater than 0.99. Back‐calculated concentrations for all calibration standards were within ±15% of nominal values (±20% at LLOQ) in all matrices (Table S1). The LLOQ was as low as 0.06 ng/mL in mouse plasma, representing approximately an 80‐fold improvement in sensitivity compared with previously reported methods [20].

**TABLE 3:** Regression equations of remodelin in biological samples.

Samples	Linear range (ng/mL)	Regression equation	*R* ^2^	LLOQ(ng/mL)
Plasma	0.06–1000	*y* = 1225.456551^∗^X+96.003916	0.998	0.06
Carotid artery	0.06–1000	*y* = 2762.925044^∗^X+191.948791	0.990	0.06
Heart	0.06–1000	*y* = 2364.607621^∗^X+412.831496	0.991	0.06
Liver	0.24–2000	*y* = 31.944451^∗^X‐4.247692	0.997	0.24
Kidney	0.31–5000	*y* = 184.543776^∗^X+147.284480	0.998	0.31

Carryover was negligible across all matrices, with residual peak areas ranging from 10.77% to 14.89% of the LLOQ (plasma: 14.89%; carotid artery: 11.00%; heart: 10.77%; liver: 14.27%; kidney: 14.30%), all well below the 20% acceptance criterion.

The UPLC–MS/MS assay achieved acceptable precision and accuracy across all tested concentrations using the QC concentrations described in Section 2.4. As shown in Table S3, the intra‐day RE ranged from −6.32% to 6.03%, and the inter‐day RE ranged from −10.24% to 11.99%, while the RSDs for precision ranged from 0.04% to 9.11% for the intra‐day and 3.17% to 12.82% for the inter‐day. All RE and RSD values were well within the ±15% acceptance criteria, confirming that the established method exhibited excellent precision and reproducibility.

Recovery and matrix effect were evaluated using the corresponding QC samples described in Section 2.4. As shown in Table 4, the recovery rates were between 85.37% and 104.35% in mouse plasma and various tissues, demonstrating high and consistent extraction efficiency. The matrix effect for remodelin at three QC levels (LQC, MQC, and HQC) ranged from 86.32% to 112.19%, indicating no significant interference from the plasma matrix or other tissues. To further assess the effectiveness of IS‐mediated compensation, the internal standard‐normalized matrix factor (IS‐normalized MF) was calculated for each matrix by dividing the matrix factor of remodelin by that of MTX. As shown in Table S2, the mean IS‐normalized MF values ranged from 0.85 to 1.09 across all matrices and QC levels, with %CV values ≤ 12.83%, confirming effective IS‐mediated matrix‐effect compensation despite the retention‐time gap between MTX and remodelin.

**TABLE 4:** Matrix effect and recovery of remodelin in biological samples.

Samples	Concentration level	Nominal conc. (ng/mL)	Matrix effects (%)	Recovery
				Mean ± SD (%)
Plasma	LQC	0.24	102.6	85.37 ± 1.92
	MQC	15.62	100.1	102.84 ± 0.33
	HQC	500	101.0	100.69 ± 0.63

Carotid artery	LQC	0.24	112.19	99.24 ± 0.88
	MQC	15.62	103.58	96.80 ± 1.17
	HQC	500	102.94	98.29 ± 0.11

Heart	LQC	0.24	93.02	97.13 ± 5.82
	MQC	15.62	105.49	95.59 ± 2.72
	HQC	500	103.59	98.28 ± 1.35

Liver	LQC	0.488	91.11	86.19 ± 1.72
	MQC	15.625	97.87	95.90 ± 6.13
	HQC	1000	86.32	104.35 ± 6.13

Kidney	LQC	0.61	104.57	91.33 ± 2.28
	MQC	39.062	87.91	97.43 ± 6.91
	HQC	2500	104.07	101.05 ± 1.21

The stability of remodelin in plasma and various tissues was comprehensively evaluated under different storage and processing conditions, and the data were detailed in Table 5. The precision (RSD) and accuracy (RE) values in multiple biological samples met the established bioassay criteria under different experimental conditions, suggesting adequate stability under the validated conditions. It is noteworthy that the chemical structure of remodelin contains a hydrazone bond, which is labile under acidic conditions. Therefore, the stability of the samples in this study was validated for up to 7 days at −40°C, which covered the maximum storage duration of actual study samples before analysis. Stability appeared to be matrix‐ and concentration‐dependent, with lower concentrations in plasma and kidney showing greater variability under prolonged storage. Furthermore, a sample randomly selected from the study was used to assess repeatability and stability. Repeatability was validated by evaluating the RSD of the peak areas from five consecutive injections of the samples (*n* = 5). Stability was evaluated by analyzing the sample in triplicate (*n* = 3) at 0, 2, 4, 8, 12, and 24 h under ambient conditions. The obtained RSD values were 0.15% for repeatability and 5.07% for stability, indicating that the processed samples retained their stability characteristics for a certain period.

**TABLE 5:** Stability of remodelin in biological samples under different storage conditions.

Samples	Concentration (ng/mL)	4 h, room temperature		24 h, −4°C		Three freeze‐thaw cycles		7 days, −40°C	
		RE (%)	RSD (%)	RE (%)	RSD (%)	RE (%)	RSD (%)	RE (%)	RSD (%)
Plasma	LQC	12.31	12.75	11.82	9.90	9.51	10.01	11.66	9.24
	MQC	7.74	10.65	7.11	8.13	6.22	13.06	6.51	10.30
	HQC	2.45	2.61	3.18	2.36	3.70	9.01	6.89	6.85

Carotid artery	LQC	0.65	3.02	−0.42	11.68	−0.48	9.56	−0.72	9.56
	MQC	0.33	0.88	−0.53	3.23	0.26	3.29	2.07	3.35
	HQC	2.17	3.51	7.47	7.32	8.50	6.39	11.09	6.39

Heart	LQC	−0.01	2.16	−0.25	3.73	−0.11	5.04	−0.21	5.04
	MQC	0.73	2.82	2.43	4.67	4.07	5.45	3.33	5.45
	HQC	2.47	3.71	3.24	3.21	1.81	4.44	5.06	4.44

Liver	LQC	2.34	2.56	1.96	2.74	6.02	7.31	10.64	7.31
	MQC	1.62	1.78	5.53	6.14	5.65	5.45	12.79	5.45
	HQC	4.27	5.87	5.77	5.72	4.75	4.99	9.32	4.99

Kidney	LQC	−11.72	8.48	−5.57	7.53	−13.97	14.82	−11.73	11.93
	MQC	11.30	9.89	10.22	8.65	14.18	9.67	7.06	9.67
	HQC	−2.69	4.52	−1.82	4.98	−0.32	5.42	−6.44	5.42

### 3.3. Quantitative Analysis of Remodelin in Biological Samples

**FIGURE 3:**
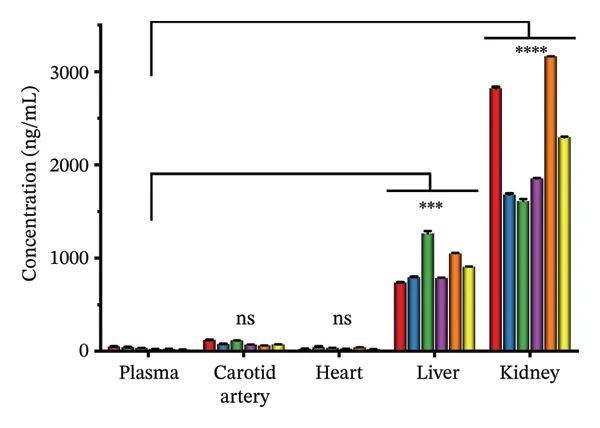
Biodistribution of remodelin in mouse plasma and tissues (carotid artery, heart, liver, and kidney) at 1 h after intraperitoneal administration (5 mg/kg). Data are shown as mean ± SD (*n* = 6 per group). ^∗∗∗^ *p* < 0.001, ^∗∗∗∗^ *p* < 0.0001 vrersus plasma; ns, not significant (one‐way ANOVA with Tukey’s post hoc test).

The tissue distribution of remodelin was examined at 1 h postdose as a proof‐of‐concept application, and the following data were presented as preliminary observations. All data were presented as mean ± SD (*n* = 6). The biodistribution of remodelin in plasma, carotid artery, heart, liver, and kidney following intraperitoneal administration was shown in Figure 3. At this single time point, the concentrations displayed a tissue‐specific pattern, with descending concentrations observed in kidney, liver, and carotid artery and comparable levels in heart and plasma. Statistical analysis using one‐way ANOVA followed by Tukey’s post hoc test revealed significantly higher remodelin levels in the liver (^∗∗∗^ *p* < 0.001) and kidney (^∗∗∗∗^ *p* < 0.0001) compared with plasma, whereas levels in the carotid artery and heart were not significantly (ns) different from plasma (both ns). These findings supported a preferential accumulation in metabolic organs, with particularly marked levels in the liver and kidney. The preferential accumulation of remodelin in the liver and kidney is consistent with its physicochemical properties as a small lipophilic molecule, which favors hepatic uptake via passive diffusion or transporter‐mediated processes, followed by biliary or renal elimination [30]. The moderate levels detected in the carotid artery and heart indicate adequate peripheral tissue penetration, supporting its potential role in ameliorating vascular remodeling and maintaining nuclear integrity in cardiovascular tissues [11]. This distribution pattern aligns with previous observations for other NAT10 inhibitors and structurally related small molecules, which also show preferential accumulation in metabolic and excretory organs. From a pharmacodynamic perspective, the enrichment in the liver and kidney is of particular interest, as these organs are central to NAT10‐mediated pathological processes, including hepatic fibrosis and renal injury [15, 21]. These findings provide a rationale for further absorption, distribution, metabolism, and excretion (ADME) studies to fully elucidate the underlying mechanisms.

### 3.4. Ultrastructure of Cells

**FIGURE 4:**
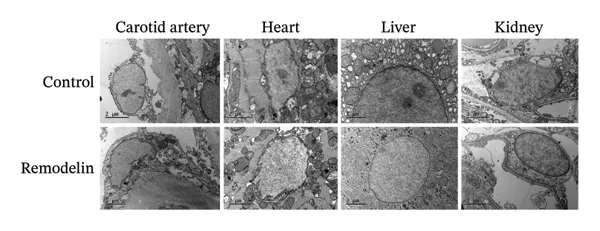
Representative TEM images of different tissues from mice treated with remodelin or DMSO after 28 days.

The results of ultrastructural analysis via TEM were presented in Figure 4. The vascular endothelial nuclei of the carotid artery receiving remodelin injection appeared intact, with preserved elastic membrane structure. The presence of cytoplasmic vacuolization in the cells was likely due to the wire‐induced carotid artery injury prior to drug administration. In contrast, cells receiving DMSO injection exhibited edematous cytoplasm and mitochondria, accompanied by signs of necrosis and shedding. In heart tissue, both nuclear and mitochondrial structures in the treatment group remained intact, whereas significant disruption of mitochondrial architecture was noted in the control group. Similarly, treated liver tissue demonstrated well‐preserved nuclei and mitochondria; conversely, dilation of the rough endoplasmic reticulum was evident in control samples. In the kidney following remodelin injection, cellular filtration barriers were preserved with patent capillaries and normal podocyte nuclei, whereas perinuclear spaces were dilated in controls. Collectively, these findings indicated that remodelin had therapeutic and protective effects.

## 4. Conclusion

This study established a simple, sensitive, and reliable UPLC–MS/MS method for the quantitative analysis of remodelin in biological samples. The method was comprehensively validated in mouse plasma and various tissues, with all parameters—including specificity, calibration linearity, LLOQ, precision, accuracy, recovery, matrix effect, and stability—meeting the predefined bioanalytical acceptance criteria, thereby confirming its robustness for preclinical studies investigating the concentration of remodelin. Subsequently, the analytical method was successfully applied to quantify remodelin concentrations in mouse plasma, carotid artery, heart, liver, and kidney at 1 h after administration. At this time point, the concentrations of remodelin in the liver and kidney were significantly higher compared to plasma and other tissues. It should be noted that these findings are preliminary and limited to the single time point examined; comprehensive time‐course studies would be valuable for a more complete understanding of remodelin’s disposition and the underlying mechanisms of its tissue‐specific accumulation. Importantly, TEM analysis revealed that remodelin treatment maintained the structural integrity of nuclear and mitochondrial membranes, with intact and well‐defined cellular architecture compared with the DMSO‐treated control group. The combined findings from UPLC–MS/MS quantification and TEM ultrastructural analysis provided preliminary evidence that remodelin may exert protective effects, highlighting its potential for further pharmacodynamic evaluation.

## Author Contributions

Litao Wang: conceptualization; methodology; validation; formal analysis; writing–original draft; project administration. Quanyu Liu: writing–original draft; project administration; funding acquisition. Cheng Yu: writing–original draft; funding acquisition. Yu Zhong: methodology; writing–original draft. Minxia Wu: software; investigation. Xi Lin: validation. Yan Hu: conceptualization; writing–review and editing; project administration; funding acquisition.

## Funding

The authors acknowledge the financial assistance from the Natural Science Foundation of Fujian Province (Grant number: 2025J01855), the Start‐up Fund for Scientific Research of Fujian Medical University (Grant number: 2023QH1015), Scientific Research Start‐up Fund for High‐level Talents of Fujian Health College (Grant number: MWY2023‐5‐03), and Excellent Young Scholars Cultivation Project of Fujian Medical University Union Hospital (Grant number: 2022XH038).

## Disclosure

A previous version of this manuscript was deposited as a preprint on SSRN: https://papers.ssrn.com/sol3/papers.cfm?abstract_id=5869490.

## Ethics Statement

All animal experiments were conducted in accordance with the guidelines of the Experimental Animal Ethics Committee of Fujian Medical University and were approved by this committee (approval number: IACUC FJMU 2022‐0704).

## Conflicts of Interest

The authors declare no conflicts of interest.

## Supporting Information

Additional supporting information can be found online in the Supporting Information section.

## Supporting information


**Supporting Information 1** This section contains three supporting tables (Tables S1–S3) providing detailed validation data for the UPLC–MS/MS method. Table S1 presents the back‐calculated concentrations of remodelin calibration standards across all biological matrices (plasma, carotid artery, heart, liver, and kidney), demonstrating acceptable accuracy at all levels. Table S2 summarizes the internal standard‐normalized matrix factors (IS‐normalized MF) at three quality control levels, confirming effective matrix‐effect compensation. Table S3 reports the intra‐day and inter‐day accuracy and precision for remodelin in each matrix, with all values within the predefined acceptance criteria. These supplementary data support the validation results presented in the main text and are referenced in Section 3.2.

## Data Availability

The data used to support the findings of this study are included within the article.
