# Supplementary material for: Development and Validation of an UPLC–MS/MS Method for Remodelin Quantification in Mouse Plasma and Tissues: Application to Biodistribution and Ultrastructural Assessment
Source: J Anal Methods Chem. 2026 Aug 24;2026:9654340. doi: 10.1155/jamc/9654340 (PMC13501163; doi:10.1155/jamc/9654340)
Supplement: Supplementary file 1 [file JAMC-2026-9654340-s001.zip › Table S1.docx]

**Table S1**

| Samples | Level | Nominal (ng/mL) | Mean back-calculated (ng/mL) | Accuracy (%) |
| --- | --- | --- | --- | --- |
| Plasma | 1 | 0.061 | 0.058 | 96.7 |
|  | 2 | 0.49 | 0.46 | 93.9 |
|  | 3 | 1.95 | 2.14 | 109.7 |
|  | 4 | 3.91 | 4.21 | 107.7 |
|  | 5 | 31.25 | 31.43 | 100.6 |
|  | 6 | 62.5 | 62.88 | 100.6 |
|  | 7 | 250 | 265.29 | 106 |
|  | 8 | 1000 | 955.55 | 95.55 |
| Carotid artery | 1 | 0.061 | 0.066 | 108.19 |
|  | 2 | 0.49 | 0.425 | 87.04 |
|  | 3 | 1.95 | 1.90 | 97.43 |
|  | 4 | 3.91 | 3.62 | 93.57 |
|  | 5 | 31.25 | 31.49 | 100.76 |
|  | 6 | 62.5 | 68.61 | 109.77 |
|  | 7 | 250 | 280.67 | 112.27 |
|  | 8 | 1000 | 915.26 | 91.52 |
| Heart | 1 | 0.061 | 0.056 | 91.80 |
|  | 2 | 0.49 | 0.44 | 90.78 |
|  | 3 | 1.95 | 1.99 | 102.41 |
|  | 4 | 3.91 | 4.19 | 107.43 |
|  | 5 | 31.25 | 35.60 | 113.92 |
|  | 6 | 62.5 | 66.97 | 107.15 |
|  | 7 | 250 | 228.2 | 91.28 |
|  | 8 | 1000 | 977.71 | 97.77 |
| Liver | 1 | 0.24 | 0.25 | 104.16 |
|  | 2 | 0.97 | 1.02 | 105.15 |
|  | 3 | 1.95 | 2.10 | 107.69 |
|  | 4 | 7.81 | 8.38 | 107.30 |
|  | 5 | 31.25 | 30.74 | 98.37 |
|  | 6 | 125 | 119 | 95.2 |
|  | 7 | 500 | 486 | 97.2 |
|  | 8 | 2000 | 2004 | 100.2 |
| Kidney | 1 | 0.31 | 0.35 | 112.90 |
|  | 2 | 1.22 | 1.29 | 105.74 |
|  | 3 | 2.44 | 2.56 | 104.92 |
|  | 4 | 9.76 | 10.88 | 111.47 |
|  | 5 | 78.125 | 68.60 | 87.81 |
|  | 6 | 312.5 | 288.95 | 92.46 |
|  | 7 | 1250 | 1324.43 | 105.95 |
|  | 8 | 5000 | 4934.17 | 98.68 |
