# Supplementary material for: Development and Validation of an UPLC–MS/MS Method for Remodelin Quantification in Mouse Plasma and Tissues: Application to Biodistribution and Ultrastructural Assessment
Source: J Anal Methods Chem. 2026 Aug 24;2026:9654340. doi: 10.1155/jamc/9654340 (PMC13501163; doi:10.1155/jamc/9654340)
Supplement: Supplementary file 1 [file JAMC-2026-9654340-s001.zip › Table S2.docx]

**Table S2**

| Samples | Concentration level | Nominal Conc. （ng/mL） | IS‑normalized MF  (mean ± SD) | CV (%) |
| --- | --- | --- | --- | --- |
| Plasma | LQC | 0.24 | 0.86±0.04 | 5.17 |
|  | MQC | 15.62 | 1.09±0.01 | 0.73 |
|  | HQC | 500 | 0.99±0.01 | 0.17 |
| Carotid artery | LQC | 0.24 | 0.89±0.03 | 3.28 |
|  | MQC | 15.62 | 1.04±0.02 | 2.22 |
|  | HQC | 500 | 1.03±0.01 | 0.77 |
| Heart | LQC | 0.24 | 0.89±0.11 | 12.83 |
|  | MQC | 15.62 | 1.05±0.02 | 2.29 |
|  | HQC | 500 | 1.03±0.03 | 2.83 |
| Liver | LQC | 0.488 | 0.85±0.04 | 4.95 |
|  | MQC | 15.625 | 0.99±0.02 | 1.97 |
|  | HQC | 1000 | 0.97±0.01 | 0.78 |
| Kidney | LQC | 0.61 | 1.05±0.07 | 7.29 |
|  | MQC | 39.062 | 0.88±0.02 | 2.23 |
|  | HQC | 2500 | 1.02±0.04 | 3.53 |
