# Supplementary material for: Development and Validation of an UPLC–MS/MS Method for Remodelin Quantification in Mouse Plasma and Tissues: Application to Biodistribution and Ultrastructural Assessment
Source: J Anal Methods Chem. 2026 Aug 24;2026:9654340. doi: 10.1155/jamc/9654340 (PMC13501163; doi:10.1155/jamc/9654340)
Supplement: Supplementary file 1 [file JAMC-2026-9654340-s001.zip › Table S3.docx]

**Table S3**

| Samples | Concentration level | Nominal Conc. （ng/mL） | Accuracy(%RE) | | Precision(%RSD) | |
| --- | --- | --- | --- | --- | --- | --- |
|  |  |  | Intra-day | Inter-day | Intra-day | Inter-day |
| Plasma | LQC | 0.18 | 6.03 | -5.85 | 8.16 | 10.06 |
|  | MQC | 500 | 0.43 | 11.99 | 0.85 | 5.14 |
|  | HQC | 750 | -6.32 | -9.08 | 0.65 | 3.17 |
| Carotid artery | LQC | 0.18 | -2.02 | -10.24 | 8.46 | 10.72 |
|  | MQC | 500 | 0.63 | 1.23 | 1.03 | 5.24 |
|  | HQC | 750 | 2.24 | -1.87 | 4.31 | 7.02 |
| Heart | LQC | 0.18 | -1.91 | -9.5 | 9.11 | 12.82 |
|  | MQC | 500 | 1.46 | 2.04 | 0.72 | 4.94 |
|  | HQC | 750 | 1.49 | -1.33 | 1.15 | 3.59 |
| Liver | LQC | 0.75 | -3.08 | -4.07 | 6.59 | 12.26 |
|  | MQC | 1000 | 3.29 | 7.17 | 0.75 | 6.78 |
|  | HQC | 1500 | -2.91 | -4.36 | 0.37 | 5.49 |
| Kidney | LQC | 1.00 | 2.74 | 4.86 | 5.24 | 7.92 |
|  | MQC | 2500 | 4.94 | 6.23 | 0.04 | 6.12 |
|  | HQC | 3750 | 4.01 | -0.06 | 1.68 | 5.83 |
